# Asymmetric Wigner molecules in nanowire Y-junctions

**DOI:** 10.1038/s41598-022-24583-x

**Published:** 2022-11-23

**Authors:** R. Méndez-Camacho, E. Cruz-Hernández

**Affiliations:** 1 Facultad de Ciencias, Universidad Autónoma de San Luis Potosí, Av. Chapultepec 1570, Privadas del Pedregal, 78295 San Luis Potosí, S.L.P. Mexico; 2 Coordinación para la Innovación y Aplicación de la Ciencia y la Tecnología, Universidad Autónoma de San Luis Potosí, Sierra Leona 550, 78210 San Luis Potosí, S.L.P. Mexico

**Keywords:** Nanoscience and technology, Physics

## Abstract

The possibility of crystalline states of interacting electrons, known as Wigner crystals, has been intensively studied in each of the three dimensions. One-dimensional (1D) systems, however, can be interconnected forming two-dimensional (2D) lattices, being a three-terminal Y-junction (Y-J) the simplest one. Then, even when electrons in the individual branches of the Y are confined in 1D, as the Y-J is in 2D, one could expect significant differences in the crystalline state of the electron gas in a Y-J. With the recent report of fabrication of defect-free GaAs/AlGaAs Y-Js by epitaxial methods, the study of semiconductor Y-Js acquires a special relevance due to its eventual direct exploration. Here, by considering the collective electron interactions using a Yukawa-like effective potential, we explore a two-electron distribution in nanowire Y-Js by modulating its electron density via a screening parameter. We find that the electrons changes from a quasi-continuous to a Wigner molecule-like distribution when the electron density decreases in the Y-J. In bold contrast to the strict 1D case, where equidistant distributions of equal density are obtained in the Wigner regime, in the Y-J equidistant distributions of asymmetric density are induced. We also explore the effect of an external electric field acting along the Y-axis on the asymmetric distributions.

## Introduction

The formation of an ordered array of electrons, where the Coulombic interactions dominate over the thermal and Fermi energies, was first pointed out, for the case of electrons in metals, by Wigner^1^. The lattice of such ordered array of electrons, known as a Wigner crystal (or, in its finite version, a Wigner molecule (WM)), strongly depends on the dimensionality (D). The condition for the Wigner crystal formation, attained at very low electron densities (*n*), can be conveniently described by a non-dimensional parameter, the Wigner–Seitz ratio $r_s$. $r_s$ represents the average electron–electron distance in units of the effective Bohr radius or, equivalently, the ratio between the Coulombic energy and the kinetic (Fermi) energy.

In three dimensions (3D), $r_s\sim \,100$ and the body-centered cubic lattice is the one with the minimal energy configuration. In contrast to the 2D and 1D cases, the very challenging experimental conditions have hindered the 3D crystal observation. In 2D, $r_s\sim \,40$ and a hexagonal lattice is predicted and experimentally observed^2^. For 1D, $r_s$ depends on the spin coherency of the electrons forming the crystal, as has been reported in remarkable experimental works^3–5^: $r_s\sim \,5$ for coherent spin and $r_s\sim \,20$ for incoherent spin. A brief survey of the 1D crystallization can be found in Ref.^6^.

Aside from the usual symmetric lattices, there exist also anisotropic ones. For example, in 2D, due to the interplay of the electron–electron ($\text {e-e}$) interaction, the localization phenomena and the Fermi sea anisotropy, anisotropic disordered Wigner crystal has been predicted^7^ and some of its signatures has been observed^8^. In 1D, disordered zigzag configurations produced by weakening the 1D confinement and by allowing the electrons to relax in a second dimension has been also reported^9,10^. In this work we are also interested in this kind of 1D anisotropies, but instead of a weak 1D confinement, a Y-J configuration is considered.

Besides the pure theoretical interest in the Y-Js, some properties with potential practical applications from Y-Js of carbon nanotubes (CNT), III–V semiconductors and even 2D materials such as graphene and phosphorene has been also investigated. Some of these properties includes electron-wave switching^11,12^, rectification^13,14,17^, nonlinear properties^15,16^, ballistic switching^18^, spin splitting^19^, quantum logic functionalities^20,21^, discontinuous thermal conductivity at the junction of the Y-J^22^, photoresponse arising only from pairwise branch combinations where a semiconductor-like junction is made from CNT^23^, as well as coherent^24^ and ballistic transport^25,26^.

**Figure 1:**
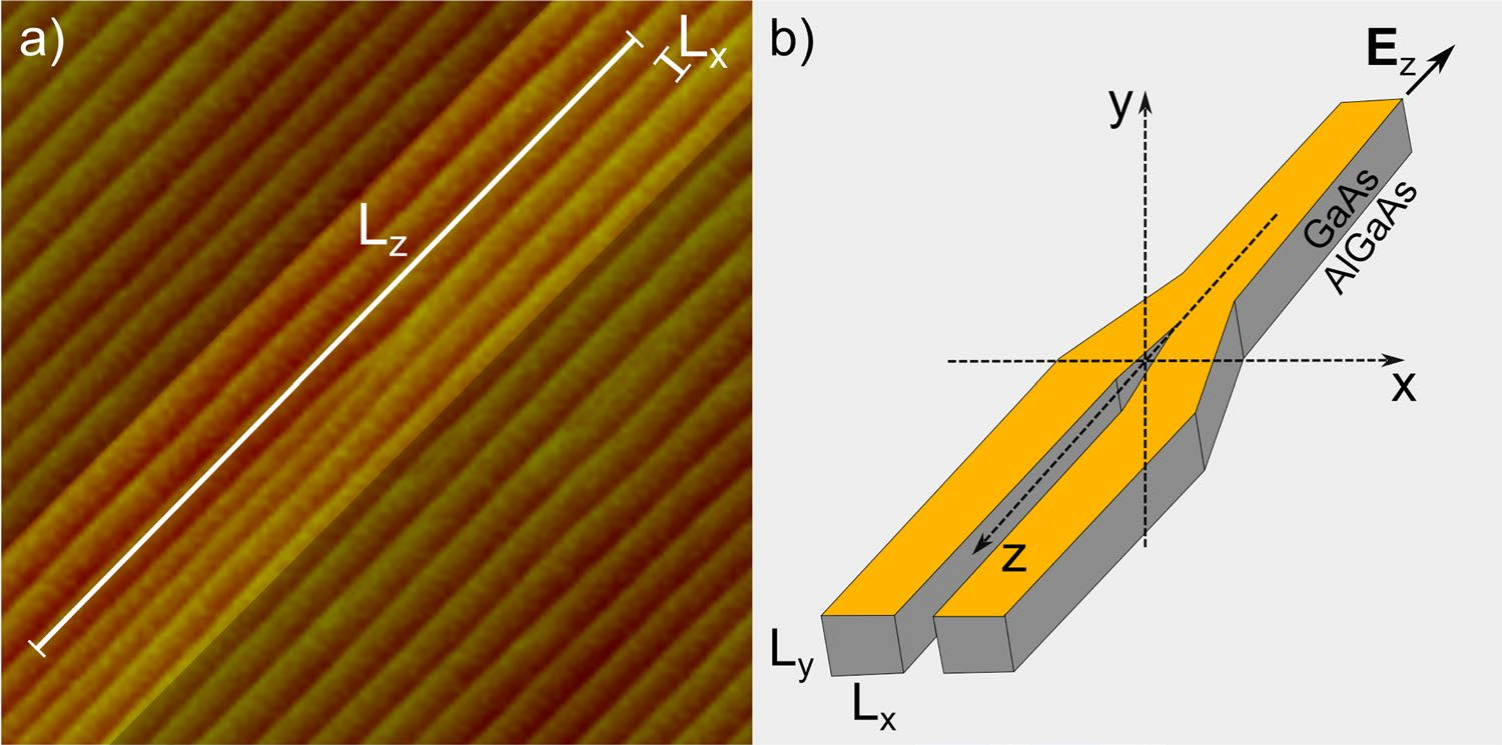
(**a**) Atomic force microscopy image of an GaAs surface of roughy 1.5 $\times$ 1.5 $\upmu \hbox {m}^2$, showing the formation of a Y-junction in a NWs network, in the Molecular Bean Epitaxy of GaAs on a (631)-GaAs high-index substrate. (**b**) Schematic illustration of the Y-junction model considered in this work.

The most studied systems are the CNT Y-Js. However, even the remarkable advances^27^ in the CNT Y-Js controlled synthesis since the first report^28^, its eventual integration in devices seems quite challenging. In contrast, the III–V compound semiconductor nanostructures grown by epitaxial techniques are much better suited for such integration^29^. Recently, our group has reported the direct self-assembly of GaAs nanowires (NWs) and Y-Js by Molecular Beam Epitaxy via energetically unstable high-index substrates^30^, then providing a valuable path toward the fabrication of III–V NWs/Y-Js networks. In Fig. 1a, we show an image taken by Atomic Force Microscopy of an array of GaAs nanowires around a Y-J, which was grown by using similar growth conditions as the reported in Ref.^30^. The model studied in the present work is based on the geometrical characteristics of this kind of Y-Js (Fig. 1b).

There exist a number of advanced formalisms to investigate the $\text {e-e}$ interaction in nanostructures, yet usually limited to one or few charge carriers and to nanostructures of the order of few nanometers^31–35^. On the other hand, to exactly study 1D electron structures such as Wigner crystals or the Friedel oscillations, one must consider the many-electron 1D interaction via the Luttinger liquid theory as well as quantum Monte Carlo or *ab initio* approaches^36–43^. However, to model micrometric long semiconductor Y-Js, considering the $\text {e-e}$ interaction of realistic doping levels ($n= 10^{16}-10^{19}$ electrons/$\hbox {cm}^3$), is an unrealizable task.

Given the difficulty of directly including the many-body forces to calculate the many-body quantum states, we opt to use a method that considers two-electron wave-functions while the presence of the remaining electrons is assumed to only affect the interaction by an electronic screening. The use of this kind of Yukawa-like potentials to address many-body problems has been extensively used in other branches of physics, such as soft matter and nuclear physics^44^. Applied to electron distributions in nanostructures, this approach has been able to correctly describe experimental results related to the Wigner molecule formation in micron-long NWs with $n= 10^{16}-10^{19}$ electrons/$\hbox {cm}^3$^45,46^, as well as to describe the effect of an external electric field (EEF) applied along the NW axis^47,48^ and the tunneling between parallel NWs^49^. In this contribution, we adapt such model to tailor the Y geometry via three interconnected NWs.

Our approach is a semi-classical one, where the finite-size two-electron (2e) WM is studied using the electron density for the infinite system as a tool to bridge the gap between the finite length and the infinite length cases. It must be noted that the theoretical methods needed to describe a finite-size 2e, by solving exactly the quantal many-body Schrödinger equation, and the infinite-length WC are quite different. It is also worth mentioning that very recently, beyond the semi-classical methods mentioned before, a Yukawa-like potential has been used to study the formation of Wigner molecules in 1D^50^ using first-order quantum phase transitions as condensations in the space of states^51^.

Finally, it must be noted that the 2e quantal WMs has been extensively studied and predicted theoretically in the past 22 years, see, e.g., the section on the 2e case in^52^. The first experimental observation of a 2e WM in an anisotropic GaAs quantum dot was reported in^53^ and the formation of WMs has been demonstrated also for the case of a contact interaction^54^. More recently, the quantal 2e-WM has been observed experimentally in GaAs double-dot quantum-computer qubits and a full theoretical explanation has also been given^55,56^.

After the introduction we present, in “Results and discussion”, GaAs/AlGaAs Y-Js ground state electronic distributions showing the quantum phase transition from extended to localized states, when *n* is lowered to $\sim \, 10^{18}$ electrons/$\hbox {cm}^3$ (and $r_s\sim \,1$). Then, we discuss the asymmetric distributions in the Y branches as compared to the symmetric ones in single NWs. Distributions from excited states and from variations of the main Y-J geometrical parameters are further on discussed. To close “Results and discussion” section, the effect of an EEF acting along the Y-J axis for some ground and excited distributions are presented. In “Methods”, a detailed derivation of the mathematical model is presented. Finally, the main conclusions are offered at the end of the manuscript in “Conclusions”.

## Results and discussion

A schematic view of our model of the GaAs/AlGaAs Y-J is provided in Fig. 1b. We consider three GaAs NWs of square cross-section (of nanometric sides $L_x= L_y \equiv L_{x,y}$), which are coupled together to form the Y geometry. The GaAs Y-J, whose axis is directed along the *z* direction, has a length of $L_{z}=$ 2 $\upmu$m and is embedded in an AlGaAs matrix. The two GaAs branches forming the Y are separated, along the *x* direction, by a distance $L_{b}$. Typical values for electronic densities in p-type extrinsic doped semiconductors were considered: $n= 10^{16}-10^{20}$ electrons/$\hbox {cm}^3$. The estimated values of $r_s$ for this range of *n* are displayed in Table 1. The minimal *n* value that the Yukawa model can manage is around $n= 10^{16}$ electrons/$\hbox {cm}^3$^46^.

**Figure 2:**
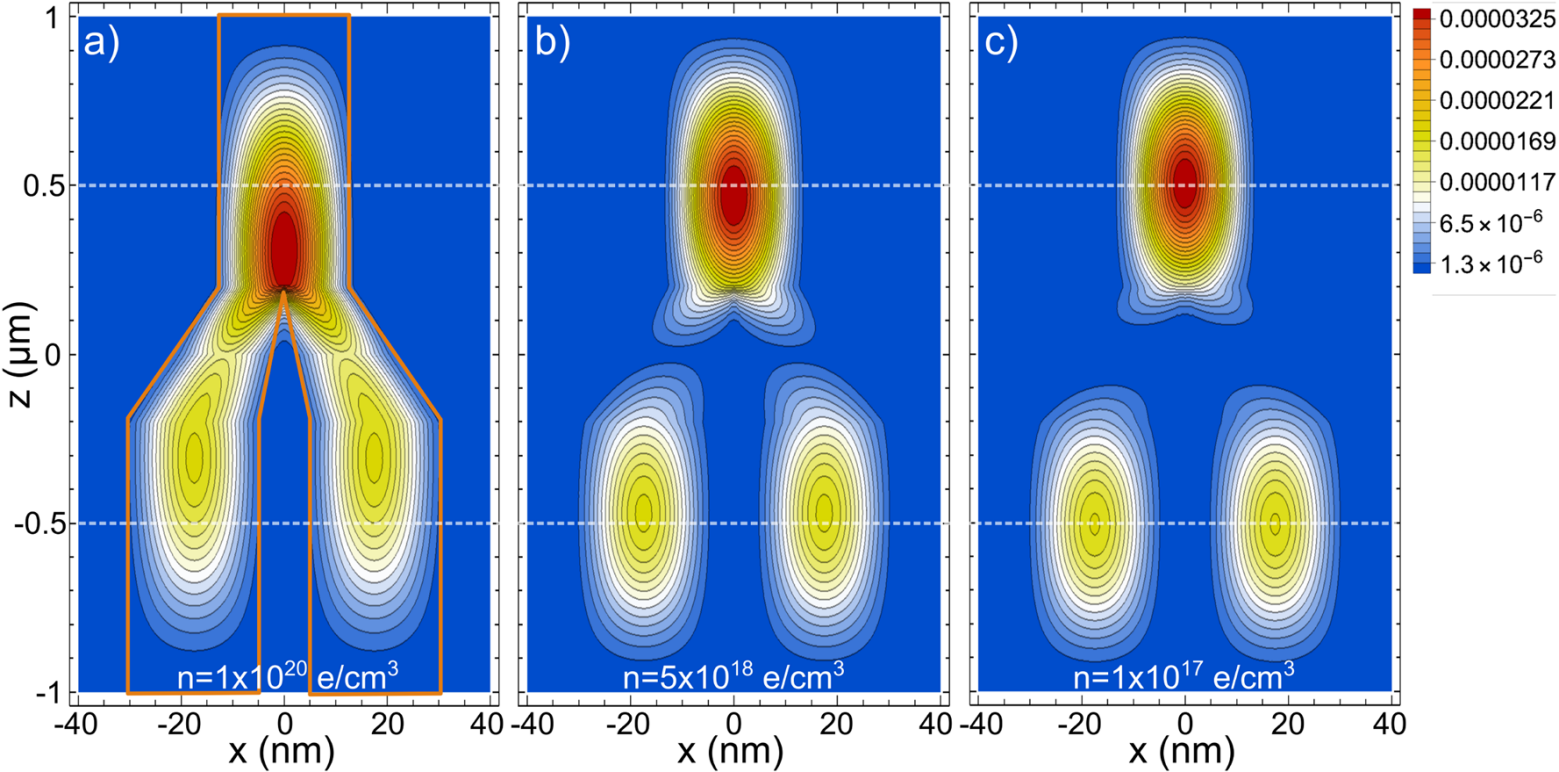
*x*–*z* projections of the two-electron distribution probability for the ground state of Y-Js of $L_{x,y}=25$ nm, $L_{b}=10$ nm, and length $L_{z}=2\,\upmu$m. Three screening parameters are considered, corresponding to densities of (**a**) 1 $\times$ 10$^{20}$ electrons/$\hbox {cm}^{3}$, (**b**) 5 $\times$ 10$^{18}$ electrons/$\hbox {cm}^{3}$, and (**c**) 1 $\times$ 10$^{17}$ electrons/$\hbox {cm}^{3}$. In (**a**), the Y-J GaAs/AlGaAs boundary is indicated. The horizontal lines are drawn to highlight the stable central positions at low densities.

A *z*-length of 2 $\upmu$m and $L_{x,y} \le 50$ nm ensure the 1D confinement in the Y-J. To avoid the tunneling between the Y branches along the *x* axis, $L_{b}$ is set to 10nm. In Fig. 2, we plot the *x*–*z* projections of the two-electron distribution probability for the ground state of Y-Js of $L_{x,y}=25$ nm, which is roughly the average size of our experimental Y-J arrays. From Fig. 2, similar to the NWs case, when the electron density is decreased from $10^{20}$ electrons/$\hbox {cm}^3$ to $10^{17}$ electrons/$\hbox {cm}^3$, a phase transition from an extended to a localized state is observed.

**Table 1:** Wigner–Seitz ratio ($r_s$) and related parameters for a Y-J of $L_{z}=2\,\upmu$m and $L_{x,y}=25$ nm.

*n* (e/$\hbox {cm}^{3}$)	Efective electrons	Average $\text {e-e}$ separation (nm)	$r_s$
1 $\times$ 10$^{16}$	59.4	45.9	4.47
1 $\times$ 10$^{17}$	594	21.5	2.1
1 $\times$ 10$^{18}$	5940	10	0.97
5 $\times$ 10$^{18}$	29,700	5.85	0.57
1 $\times$ 10$^{19}$	59,400	4.64	0.45
1 $\times$ 10$^{20}$	594,000	2.15	0.21

In NWs, equidistant localized state distributions are a signature of the WM formation. However, in contrast to the NWs, in the Y-J such localizad states are not symmetrical along the branches in the *z* axis (because the individual top state is denser than the two lower distributions, see Fig. 2c). Still, if one integrates over the three localized distributions, it turn out that each lower distribution has the same value and that both are one-half of the value of the top distribution. That is, is like the lower electron was equally shared between the two bottom branches while the top one remains unchanged. Charge fractionalization is an exciting phenomenon predicted in 1D for the spinless Luttinger model^57,58^ that has been experimentally observed in NWs^59^ and, more recently, in quantum Hall Y-Js^60^. It is noteworthy that the approach we employ, even rough, is able to reproduce such fractionalization in semiconductor nanometric Y-Js.

**Figure 3:**
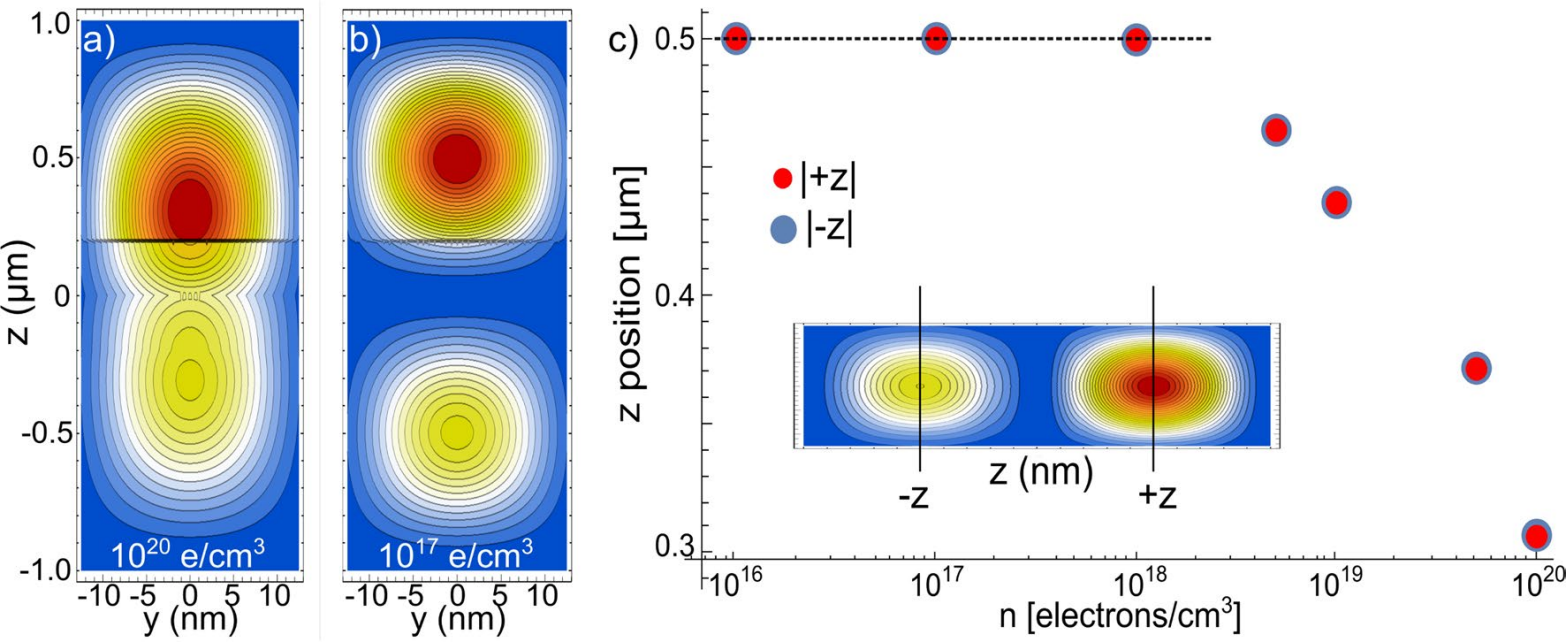
(**a**) *y*–*z* projections of the electron distribution probability corresponding to Fig. 2a. (**b**) *y*–*z* projections corresponding to Fig. 2c. (**c**) *z*-position of the left(blue)- and right(red)-central distributions, defined as schematized in the inset, for different *n*. When $|\pm \, z| =0.5$, a Wigner-like molecule distribution is obtained.

A different perspective, the *y*–*z* projection, of the phase transition shown from Fig. 2a–c, are presented in Fig. 3a–b, respectively. In Fig. 3c, we plot the $|\pm \, z|$ central positions of the two-fold density distribution (in the inset it is schematized such *z* positions), as a function of *n*, for a Y-J of $L_{x,y}=25$ nm, $L_{b}=10$ nm, and length $L_{z}=2\,\upmu$m. When $|\pm \, z| =0.5\,\upmu$m, a equidistant (Wigner-like) distribution is reached. As can be seen, for such Y-J the localized sates are generated for $n \le 1\times 10^{18}$ electrons/$\hbox {cm}^{3}$, or equivalently (see Table 1), to $r_s \sim \, 1$ or larger.

**Figure 4:**
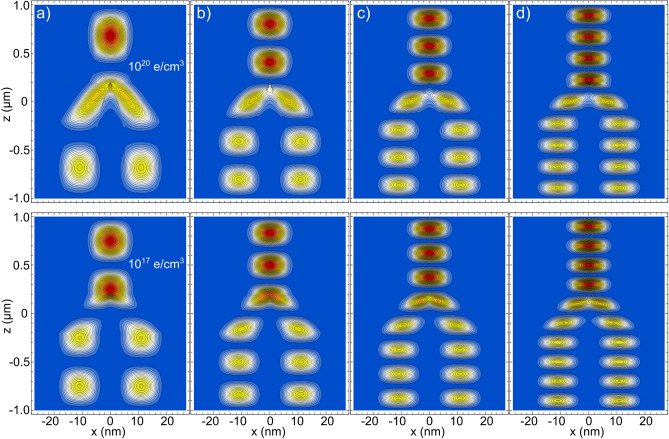
Electronic density probability corresponding to the (**a**) first-, (**b**) second-, (**c**) third- and (**d**) fourth-excited states for Y-junctions of $L_{x,y}=12$ nm, $L_{b}=$10 nm, and $L_{z}=2\,\upmu$m. The top row corresponds to a density of 1 $\times$ 10$^{20}$ electrons/$\hbox {cm}^{3}$ and the bottom row to a density of 1 $\times$ 10$^{17}$ electrons/$\hbox {cm}^{3}$.

We plot in Fig. 4, the first four *z* excited states of a Y-J of $L_{x,y}=12$ nm, for two representative values of *n*. The importance and properties of the excitations of a quantal 2e-WM have been investigated also in^61^. As the energy difference between the *x* and *y* energy levels is very large ($\sim$ meV) as compared with the *z* component ($\sim \,\upmu$eV), we only consider the *z* excited state while the *x* and *y* are kept in the ground state. We consider the excited states for two main reasons: (1) since these levels are quite close, it must be easily populated by a weak external perturbation and, (2) as one of the well know results for WM in both the Luttinger liquid theory and the experimental observations is that there are a number of well separated peaks that equals the number of electrons in the system. By allowing us this analogy, we can get some valuable grasp of the many-electron distribution in the Y-J by examining the excited states (which is particularly useful when the effect of an EEF is considered).

In Fig. 4, we can observe interesting features at the Y-intersection, where the two-electron distribution interacts in a more complex way than in the other parts of the Y. It can also be noted that the asymmetry in the density remains, being larger in the individual top branches than the two lower ones. Also it can be seen that, in contrast to the ground state, for both densities there are localized states (which is according to previous results on NWs). We also note that with the modification of *n*, it is produced a slight difference in the number of individual peaks.

**Figure 5:**
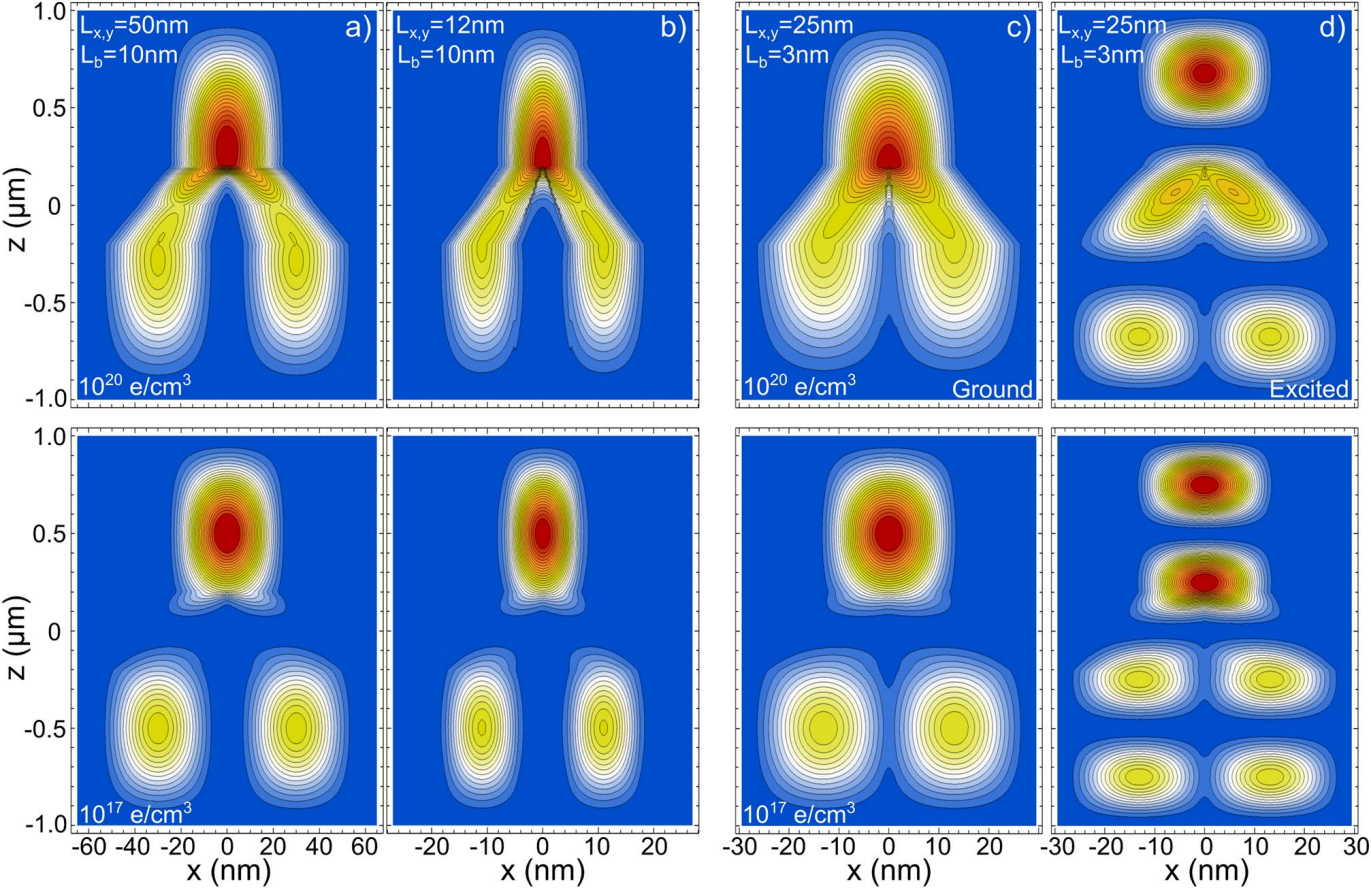
Electronic distributions from Y-Js of different sizes: column (**a**) $L_{x,y}=50$ nm, $L_{b}=$ 10 nm, and $L_{z}=2\,\upmu$m; column (**b**) $L_{x,y}=12$ nm, $L_{b}=$ 10 nm, and $L_{z}=2\,\upmu$m; columns (**c**,**d**) $L_{x,y}=25$ nm, $L_{b}=$ 3 nm, and $L_{z}=2\,\upmu$m. Only in column (**c**) are presented excited states. The top row corresponds to a density of 1 $\times$ 10$^{20}$ electrons/$\hbox {cm}^{3}$ and the bottom row to a density of 1 $\times$ 10$^{17}$ electrons/$\hbox {cm}^{3}$.

Before dealing with the effect of an EEF on the Y-J distributions, we briefly present two other parameter variations in Fig. 5, $L_{x,y}$ and $L_{b}$. In order to observe the influence of the cross-section size on the electronic distribution, in Fig. 5a–b we plot the ground state distributions for relative large ($L_{x,y}=50$ nm) and relative small ($L_{x,y}=12$ nm) Y-Js for two representative *n* values. As expected, for $L_{x,y}=12$ nm, the stronger confinement forces the electronic charge to redistributes along the Y-J. Also, for the smaller cross section the electronic tunneling outside the Y-J is more pronounced (more visible at the intersection).

As we are considering transversal finite barriers along the *x*-axis, our model also allows to observe electronic tunneling between the Y-branches when $L_{b}$ is small enough. In Fig. 5c–d the ground and first excited states, respectively, of a Y-J with a very small $L_{b}=$3 nm are presented. Electronic tunneling can be observed for this small separation between the lower branches of the Y, quite similar to the recently reported tunneling between parallel NWs^49^. Due to the symmetric charge distributions along the z axis of the Y-J, the overlap of electron wavefunctions only happens between adjacent electronic distributions (along the *x*-axis), being stronger where the distribution is denser. This lateral tunneling between the Y-J branches, connecting electronic independent distributions, is an interesting feature that could be of practical and theoretical interest.

**Figure 6:**
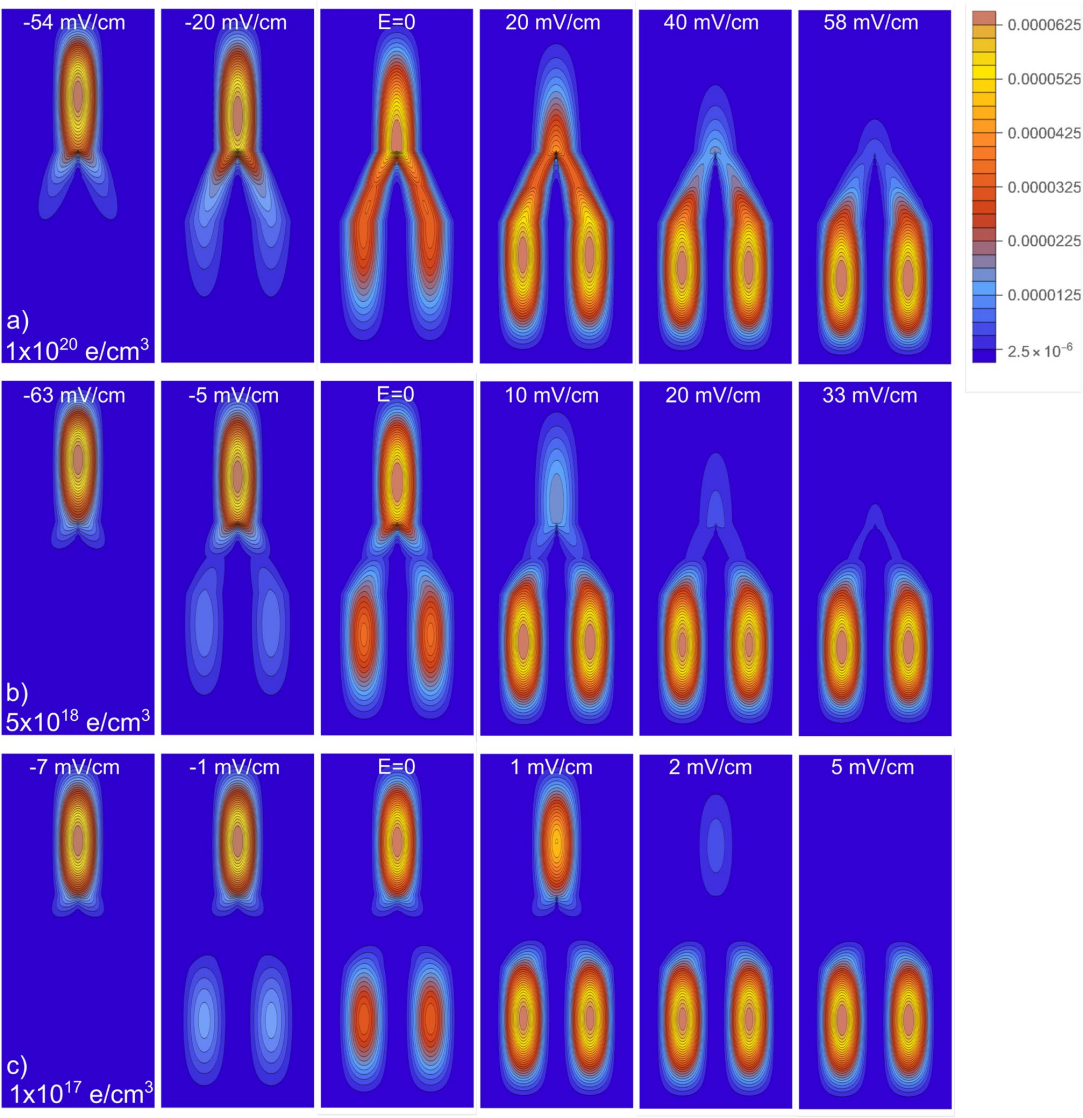
Ground state *x*–*z* projections of the charge distribution probability Y-Js of $L_{x,y}=25$ nm, $L_{b}=10$ nm, and length $L_{z}=2\,\upmu$m. Density *n* varies from row to row: (**a**) 1 $\times$ 10$^{20}$ electrons/$\hbox {cm}^{3}$, (**b**) 5 $\times$ 10$^{18}$ electrons/$\hbox {cm}^{3}$, and (**c**) 1 $\times$ 10$^{17}$ electrons/$\hbox {cm}^{3}$. The electric field magnitude applied along the *z*-axis is indicated in each figure. *x* and *z* scales are the same as the ones in Fig. 2.

**Figure 7:**
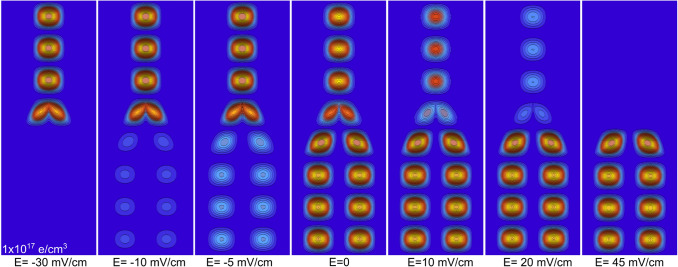
*x*–*z* projections of the charge distribution probability for the third-excited state of a Y-J of $L_{x,y}=25$ nm, $L_{b}=10$ nm, length $L_{z}=2\,\upmu$m, and *n*=5 $\times$ 10$^{17}$ electrons/$\hbox {cm}^{3}$. The electric field magnitude applied along the *z*-axis is indicated in each figure. *x* and *z* scales are the same as the ones in Fig. 2.

The experimental study of the WM formation is usually quite challenging and a number of different ingenious experiments has been proposed^3–5^. However, even the particularities of each experimental set up, the use of an external perturbation such as a bias voltage or EEF is always needed. Similarly, in semiconductor nanostructure devices one need to apply a voltage difference or EEF between the device terminals to activate some useful property. Then, in order to elucidate future experimental studies, in the rest of this contribution we focus on the effect that an EEF has on the Y-Js electronic distributions. In the following plots, the EEF magnitudes were chosen to trigger noticeable modifications in the electronic distribution.

In Fig. 6, we plot the *x*–*z* projections of the distribution probability for the ground state of Y-Js with the same parameters as the plotted in Fig. 1 (which corresponds, in Fig. 6, to the column with EEF = E = 0). The redistribution on the electronic density due to an EEF directed along both, the positive and negative directions of the *z* axis are displayed. First of all, we can observe from Fig. 6 that our model is able to redistribute the charge along the two sides of the Y-J as one could expect. Second, we can see that the asymmetric charge distribution at E = 0 can be modified (as expected), by conveniently tuning the EEF. The latest is important to support the main result of this contribution. That is, we can say that the formation of an asymmetric WM in Y-Js at low densities, predicted by our model, is trustworthy as long as this model is also able to symmetrically redistribute the charge density when an adequate EEF is applied.

The particular way that the electron density is moved along *z* when the electronic states are quasi continuous or localized are quite similar to the case of single NWs^48^, including the WM stability when the EEF is applied at low densities. This stability means that the place where the distributions are centered (the $|\pm \, z|$ positions in Fig. 3c), are not modified by the application of the EEF, even when the density does move from one localized distribution to the other. In NWs, this transfer occurs from one distribution to other; however, in the Y-Js the transfer occurs from one distribution to two distributions (for an EEF directed along $+z$) or from two distributions to one distribution (for an EEF directed along $-z$).

Finally, in Fig. 7 the *x*–*z* projections for the third-excited state distribution, corresponding to a Y-J with the same parameters as the one in Fig. 6c, are plotted for different EEF magnitudes and *z*-orientation. Aside from the similar information as obtained from the excited states plotted in Fig. 4, we can observe two additional features: (1) the EEF magnitude needed to almost completely empty the single branch of the Y (45 mV/cm) is significantly larger than the needed to empty the two lower branches (30 mV/cm), which could be connected with some kind of rectification property, and (2) the population of each localized state in the single top branch (or the two low branches), remains at the same value even when, as a whole, it is modified by the EEF. That is, there is not a density gradient in the localized states in each part of the Y: the gradual change only occurs in the localized state at the very junction of the three branches.

We would like to highlight that, given on the one hand the possibility to induce an array of well separated charge distributions, in pinning positions, which can be populated or depopulated by means of an external gate and, on the other hand, the controlled self-assembly of NWs and Y-Js arrays as the one shown in Fig. 1; together it could give place to investigate new physical phenomena or practical applications involving 2D architectures of 1D confined systems.

## Methods

In this section, we follow the numerical solution of the Schrödinger equation for two electrons interacting via a Yukawa-like effective potential^46^, which was originally used to study individual semiconductor NWs. We also consider the modifications required to incorporate an external electric field^48^ and arrays of multiple parallel NWs^49^. In the following, we outline the key steps to understand the adaptation we made to the reported models to tailor the Y-J geometry: (1) the derivation of the effective potential, as in reference^49^, for arrays of $1 \times 1$ (the bottom part of the Y) and $1 \times 2$ (the top part of the Y) of parallel NWs, (2) the incorporation of a longitudinal EEF^48^ and, (3) the way we manage the coupling of the three NWs.

The time-independent Schrödinger equation for a spinless two-electron wave function, $\Psi _{1,2} \equiv \Psi _{1,2} ((x_1,y_1,z_1),(x_2,y_2,z_2))$, is

1 $$\begin{aligned} \left[ -\frac{\hbar ^2}{2m_{e}^*} \nabla _{1,2} ^2 +V_{\text {eff}}\right] \Psi _{1,2} =E \Psi _{1,2} , \end{aligned}$$

where $\hbar \equiv h/2\pi$, *h* the Planck constant, $m_e^*$ the electron effective mass, and $V_{\text {eff}}$ is the effective potential. This $V_{\text {eff}}$ capture the Yukawa-like interaction between the electrons and also the finite potentials from the interfaces GaAs/AlGaAs in the *x*–*y* plane^46^. For an array of $1 \times m$ parallel NWs, $V_{\text {eff}}$ is given, at the extreme left and right AlGaAs barriers, by^48^:

2 $$\begin{aligned} V_{\text {eff}_\text {b}}= \frac{\left[ N_y \pi e \right] ^2}{8 \pi \epsilon _\text {b}} \left[ A_\text {i}^2 + B_\text {i}^2 \right] \left[ \frac{e^{-|z|\sqrt{4k_{\text {b}_x}^2+4k_y^2+\kappa ^2}}}{\sqrt{4k_{\text {b}_x}^2+4k_y^2+\kappa ^2}} \right] ,\nonumber \\ \end{aligned}$$

at the internal AlGaAs barriers,

3 $$\begin{aligned} V_{\text {eff}_\text {b}}= \frac{\left[ N_y\pi e\right] ^2}{8\pi \epsilon _\text {b}}\left[ \frac{A_\text {i}^4+B_\text {i}^4}{A_\text {i}^2B_\text {i}^2}\right] \left[ \frac{e^{-|z|\sqrt{4k_{\text {b}_x}^2+4k_y^2+\kappa ^2}}}{\sqrt{4k_{\text {b}_x}^2+4k_y^2+\kappa ^2}} \right] ,\nonumber \\ \end{aligned}$$

and, in the GaAs Y-J,

4 $$\begin{aligned} V_{\text {eff}_{\text {nw}}}= \frac{\left[ N_y\pi e\right] ^2}{64\pi \epsilon _{\text {nw}}} \frac{\left[ C_\text {j}^2+ D_\text {j}^2\right] ^2}{C_\text {j}^2 D_\text {j}^2} \left[ \frac{e^{-|z|\sqrt{4k_{\text {nw}_x}^2+4k_y^2+\kappa ^2}}}{\sqrt{4k_{\text {nw}_x}^2+4k_y^2+\kappa ^2}} \right] , \nonumber \\ \end{aligned}$$

where $\epsilon _{\text {nw/b}}=\epsilon _0\epsilon _{r_{\text {nw/b}}}$ is the absolute permittivity for each region, with $\epsilon _0$ the vacuum permittivity and $\epsilon _r$ the relative permittivity of the material ($\epsilon _{r_\text {nw}} =12.9$ for GaAs and $\epsilon _{r_\text {b}} =12.247$ for Al$_x$Ga$_{1-x}$As for the Al concentration $x=0.23$ considered in the calculations). The screening parameter $\kappa$ is given by $\sqrt{\frac{2e^2\text {n}}{\epsilon K_{B} T}}$, with *n* the electronic density, $K_B$ the Boltzmann constant, and *T* ($=300$ K) the temperature. $k_{\text {nw}_{x}}=\sqrt{\frac{2m_{\text {nw}}^*}{\hbar ^2} E_{x}}$, $k_{\text {b}_{x}}=\sqrt{\frac{2m_{\text {b}}^*}{\hbar ^2} (V_0-E_{x}})$ and $k_y=\sqrt{\frac{2m_{\text {nw}}^*}{\hbar ^2} E_{y}}$, $E_x$ and $E_y$ the eigen-energies, $V_0 = 187$ meV, $m_{\text {nw}}^* = 0.0665m_e$ for GaAs and $m_{\text {b}}^* = 0.0857m_e$ for Al$_{0.23}$Ga$_{0.77}$As ($m_e$ the electron mass), and $A_i, B_i, C_j, D_j$ and $N_y$ are the normalization constants, in which $\text {i}=1,2,3,\ldots (m+1)$, $\text {j}=1,2,3,\ldots m$. For the single Y-J considered here, $m= 1$ in the top part of the Y and $m= 2$ in the bottom part.

To plot the electronic densities in the branches of the Y-J, we solve Eq. (1) using the adequate $V_{\text {eff}}$ by using the finite difference method. At the place were the three NWs connect each other to form the Y, we slice such region into thin segments along the *z* direction. For each thin segment, we calculate $E_x$ by solving the Schrödinger equation for the two-finite wells in the *x* direction with the respective $L_b$-size modification and one finite well in the *y* direction. Then, introducing the modified $E_x$ and $E_y$ values in $V_{\text {eff}}$ for each segment we solve the Schrödinger equation in the *z* direction, with its respective $V_{\text {eff}}$, and using the separation of the wave function as $\Psi _{1,2}= \psi _x(x_1, x_2) \psi _y(y_1, y_2) \psi _z(z_1, z_2)$. Then, we plot the 2D *x*–*z* and *y*–*z* projections of the two-electron probability density via $|\psi _x(x_1, x_2)\psi _z(z_1, z_2)|^2$ and $|\psi _y(y_1, y_2)\psi _z(z_1, z_2)|^2$, respectively. In the calculations, 200 thin slices were considered.

Finally, to incorporate the effect of the EEF to the $V_{\text {eff}}$, we follow the analytical derivation reported in^48^, which must be adapted to an array of $1 \times m$ parallel NWs. As a result, the next expressions must added to $V_{\text {eff}}$ in the appropriated region.

To Eq. (2),

5 $$\begin{aligned} U_{\text {eff}_\text {b}}= 8N_y^2\pi ^2 z e E_{\text {ext}} \left[ A_\text {i}^2 + B_\text {i}^2 \right] ,\nonumber \\ \end{aligned}$$

to Eq. (3),

6 $$\begin{aligned} U_{\text {eff}_\text {b}}= 4N_y^2\pi ^2z e E_{\text {ext}} \left[ \frac{A_\text {i}^4+B_\text {i}^4}{A_\text {i}^2B_\text {i}^2}\right] ,\nonumber \\ \end{aligned}$$

and, to Eq. (4),

7 $$\begin{aligned} U_{\text {eff}_{\text {nw}}}= \frac{N_y^2\pi ^2 ze E_{\text {ext}}}{8} \frac{\left[ C_\text {j}^2+ D_\text {j}^2\right] ^2}{C_\text {j}^2 D_\text {j}^2}, \nonumber \\ \end{aligned}$$

## Conclusions

In summary, we have adapted some models reported by our group to tailor the geometry of a three-terminal semiconductor nanowire Y-junction. Such model allows the study of interacting electrons in micron-long Y-Js, exploring usual electronic densities in the experimental doping of GaAs/AlGaAs compounds. We find that the two-electron density distribution in the J-Y changes from quasi-continuous to localized states when the *n* density decreases from $\sim \,1\times$ 10$^{20}$ electrons/$\hbox {cm}^{3}$ ($r_s \sim \, 0.2$) to $\sim \,1 \times$ 10$^{18}$ electrons/$\hbox {cm}^{3}$ ($r_s \sim \, 1$). In bold contrast to the NW case, where the localized states are roughly similarly populated, in the Y-J the population in the single branch is twice the population of each of the two branches forming the Y. This asymmetry was also studied with the application of an EEF, by populating and depopulating the electronic states in both parts of the Y. Finally, we also highlight some possible uses of the NWs, Y-Js and, in general, more complex 2D networks of 1D interconnected systems.

## Data Availability

The datasets used and/or analysed during the current study available from the corresponding author on reasonable request.
